# Human Mobility and Droplet-Transmissible Pediatric Infectious Diseases during the COVID-19 Pandemic

**DOI:** 10.3390/ijerph19116941

**Published:** 2022-06-06

**Authors:** Ryusuke Ae, Yoshihide Shibata, Toshiki Furuno, Teppei Sasahara, Yosikazu Nakamura, Hiromichi Hamada

**Affiliations:** 1 Division of Public Health, Center for Community Medicine, Jichi Medical University, Yakushiji 3311-1, Shimotsuke 329-0498, Tochigi, Japan; shirouae@jichi.ac.jp (R.A.); shibata.yoshihide@gifu-nct.ac.jp (Y.S.); protozoa@jichi.ac.jp (T.S.); nakamuyk@jichi.ac.jp (Y.N.); 2 Department of Electrical and Computer Engineering, National Institute of Technology, Gifu College, 2236-2 Kamimakuwa, Motosu 501-0495, Gifu, Japan; 3 Advanced Course for Interdisciplinary Technology Development, National Institute of Technology, Gifu College, 2236-2 Kamimakuwa, Motosu 501-0495, Gifu, Japan; furunotoshiki0304@gmail.com; 4 Department of Pediatrics, Tokyo Women’s Medical University Yachiyo Medical Center, 477-96 Owada-Shinden, Yachiyo 276-0046, Chiba, Japan; 5 Department of Pediatrics, Graduate School of Medicine, Chiba University, 1-8-1 Inohana, Chuo-ku, Chiba-shi 260-8670, Chiba, Japan

**Keywords:** pediatric infectious disease, droplet infectious disease, movement of people, human mobility, social distancing, coronavirus disease-2019, pandemic

## Abstract

The study tested the hypothesis that human mobility may be a potential factor affecting reductions in droplet-transmissible pediatric infectious diseases (PIDs) during the coronavirus disease-2019 (COVID-19) pandemic mitigation period in 2020. An ecological study was conducted using two publicly available datasets: surveillance on infectious diseases collected by the Japanese government and COVID-19 community mobility reports presented by Google. The COVID-19 community mobility reports demonstrated percentage reductions in the movement of people over time in groceries and pharmacies, parks, and transit stations. We compared the weekly trends in the number of patients with droplet-transmissible PIDs identified in 2020 with those identified in the previous years (2015–2019) and assessed the correlations between the numbers of patients and percentage decreases in human mobility during 2020. Despite experiencing their peak seasons, dramatic reductions were found in the numbers of patients with pharyngoconjunctival fever (PCF) and group A streptococcal (GAS) pharyngitis after the tenth week of 2020. Beyond the 20th week, no seasonal peaks were observed in the number of patients with all PIDs identified in 2020. Significant correlations were found between the percentage decreases in human mobility in transit stations and the number of patients with hand-foot-and-mouth disease (Pearson correlation coefficient [95% confidence interval]: 0.65 [0.44–0.79]), PCF (0.47 [0.21–0.67]), respiratory syncytial virus infection (0.45 [0.19–0.66]), and GAS pharyngitis (0.34 [0.06–0.58]). The highest correlations were found in places underlying potential human-to-human contacts among adults. These findings suggest that reductions in human mobility for adults might contribute to decreases in the number of children with droplet-transmissible PIDs by the potential prevention of adult-to-child transmission.

## 1. Introduction

Severe acute respiratory syndrome coronavirus 2 (SARS-CoV-2) infection has been spreading worldwide since 2019. To address the rapid increase in patients with coronavirus disease-2019 (COVID-19) during the pandemic, many countries have carried out citywide lockdown measures to minimize the spread of the infection, resulting in great restriction on the movement of people [1,2,3,4]. In Japan, the government began to formally close schools nationwide from the tenth week in 2020 in response to the rapid COVID-19 outbreak. A nationwide State of Emergency was subsequently announced for the period encompassing the 15th–19th weeks. School closure continued until the State of Emergency was officially lifted in the 19th week. Social distancing and mask-wearing have also been required, based on increasing evidence for their effectiveness in protecting against SARS-CoV-2 infection [4,5,6,7,8,9,10,11].

Studies have shown that the number of children with common pediatric infectious diseases (PIDs) other than COVID-19 decreased in 2020 compared with previous years [12,13,14,15,16,17,18,19,20,21]. One study suggested that social distancing may have had the greatest impact on decreasing the number of children with PIDs during the SARS-CoV-2 pandemic [14]. However, to our knowledge, no studies have determined the specific potential factors contributing to the reductions in PIDs. We hypothesized that human mobility may be a potential factor affecting reductions in PIDs that can be transmitted by droplets among children. To test this hypothesis, we evaluated the associations between human mobility and trends in PIDs by using datasets from Japanese infectious disease surveillance and nationwide human mobility reports. 

## 2. Materials and Methods

### 2.1. Data Sources and Study Design

An ecological study was conducted using two publicly available datasets: (1) Japanese surveillance of infectious diseases and (2) COVID-19 community mobility reports. Institutional review board approval was not required for the study because the data did not contain individual personal information and were publicly available through websites.

The Japanese government conducts the National Epidemiological Surveillance of Infectious Diseases program and has obtained information on the diseases specified in the Infectious Diseases Control Law since 1999. The number of patients with the specific infectious diseases are updated and publicized weekly on a website presented by the National Institute of Infectious Diseases [22]. Although some infectious diseases are notifiable and reported immediately upon occurrence by every medical facility throughout Japan, common PIDs are reported by approximately 3000 sentinel medical facilities across Japan (sentinel surveillance) [23]. Common PIDs comprise respiratory syncytial virus (RSV) infection, pharyngoconjunctival fever (PCF), group A streptococcal (GAS) pharyngitis, infectious gastroenteritis, chicken pox, hand-foot-and-mouth disease (HFMD), erythema infectiosum, roseola infantum, herpangina, and mumps [23]. Among these 10 diseases, the present study focused on RSV infection, PCF, GAS pharyngitis, HFMD, and herpangina because of their nature as droplet-transmissible PIDs. Chicken pox and mumps were excluded from the analysis because specific vaccinations are available for these diseases.

COVID-19 community mobility reports provided by Google LLC are also publicly available [24]. These reports offer insights into how national human mobility has changed in response to policies aimed at combating COVID-19 in 131 countries worldwide. Google distills these insights from data on users who opted-in for “Location History” in their Google Account based on support for public health policy [25]. The datasets demonstrate the human mobility trends over time. Specifically, the datasets revealed how people visiting categorized places changed compared with the baseline days, with the data presented as percent changes. A baseline day provides a standard value for that day of the week, and represents the median value for that day over a 5-week period (3 January to 6 February 2020) [26]. The six categorized places are retail and recreation areas, groceries and pharmacies, parks, transit stations, workplaces, and residential areas. Among these categorized places, the present study focused on groceries and pharmacies, parks, and transit stations, assuming that these areas mainly reflect the movement of people. Percentage reductions in human mobility negatively become larger when the movement of people is more greatly restricted.

### 2.2. Statistical Analysis

First, we evaluated the weekly trends in the number of patients with PIDs as well as the percentage reductions in human mobility in Japan for every week in the year 2020 (52 weeks). To evaluate the differences in the trends of the number of patients with PIDs between the year 2020 and the previous years (2015–2019); the numbers of patients with PIDs identified during each year (2015 through 2019) were also presented separately. The human mobility data were originally obtained as daily data, and subsequently changed into weekly data, representing the mean values for each week. The period comprising the 10th–19th weeks is highlighted in the charts because the movement of people across Japan was largely restricted during this period due to the nationwide school closure and the State of Emergency.

Second, we evaluated the associations between the number of patients with PIDs and percentage reductions in human mobility using the Pearson correlation analysis. Scatter plots were created to determine the distributions of the associations between these variables. We further created a heat map to clarify the strengths of the correlation coefficients between variables according to the specific PIDs and categorized places. The correlation coefficients were determined to be significant when the absolute values of the lowest limit of the 95% confidence intervals (CIs) were higher than zero; statistical tests were also employed to confirm the significancy with the significance threshold set at *p* < 0.05. The 2.5 and 97.5 percentiles were used to express the 95% CIs. All analyses were performed using Python version 3.7.4 (The Python Software Foundation, 2020, Beaverton, OR, USA).

## 3. Results

**Figure 1:**
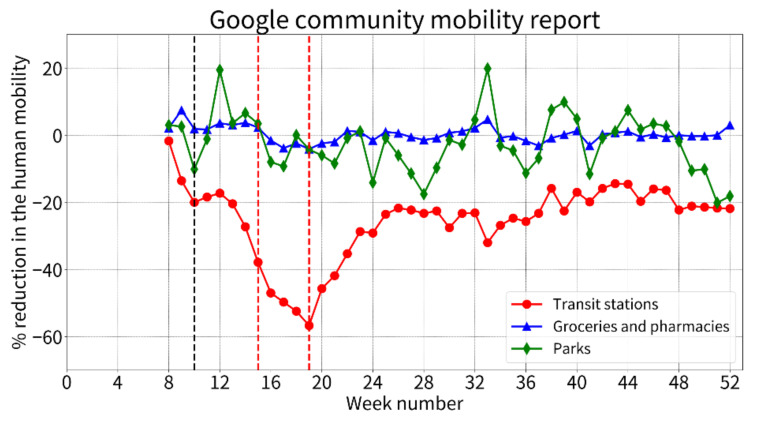
**The weekly percentage reductions in human mobility in 2020 (5th–52nd weeks).** The percentage reductions in human mobility represent the mean values for each week compared with the baseline 5-week period (3 January to 6 February 2020). The initiation of the nationwide school closure at the 10th week is highlighted by a black dashed line and the national State of Emergency from the 16th to 19th weeks is shown by the red dashed lines. The schools typically reopened after the State of Emergency was officially lifted in the 19th week.

Figure 1 shows the weekly trends in the percentage reductions in human mobility in 2020 (5th–52nd week). Compared with the standard values defined as the mean human mobility in the baseline period (1st to 5th weeks), human mobility in transit stations rapidly decreased from the 14th week, reached the lowest percentage of >50% reduction at the 19th week, rebounded toward an increase, and subsequently remained at approximately 20% reduction until the end of 2020. Human mobility in groceries and pharmacies was slightly decreased after the 14th week throughout the observation period with some variation, while that in parks remained almost unchanged.

**Figure 2:**
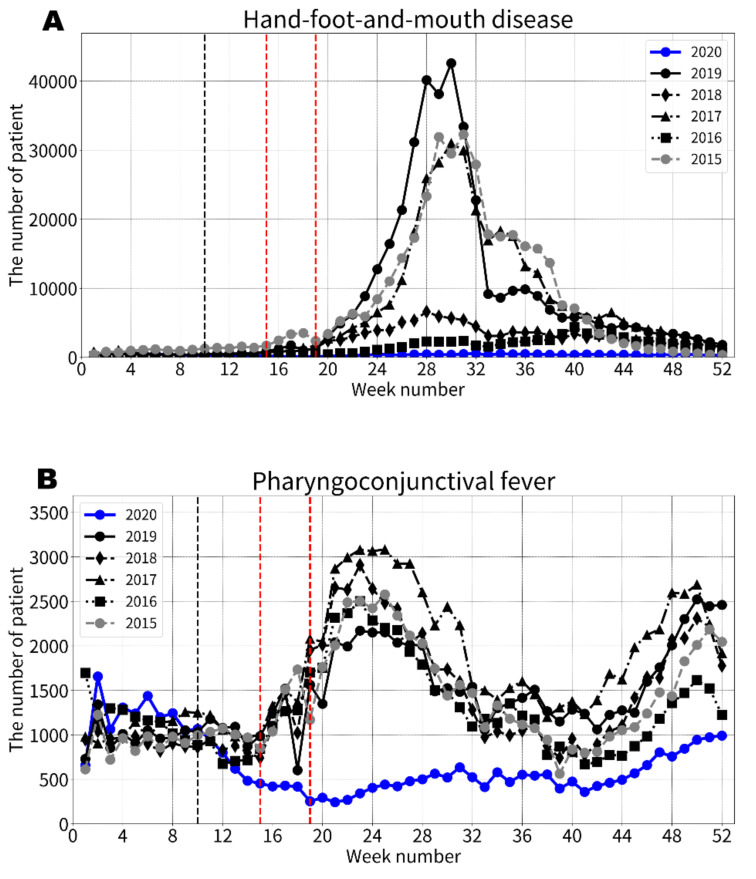

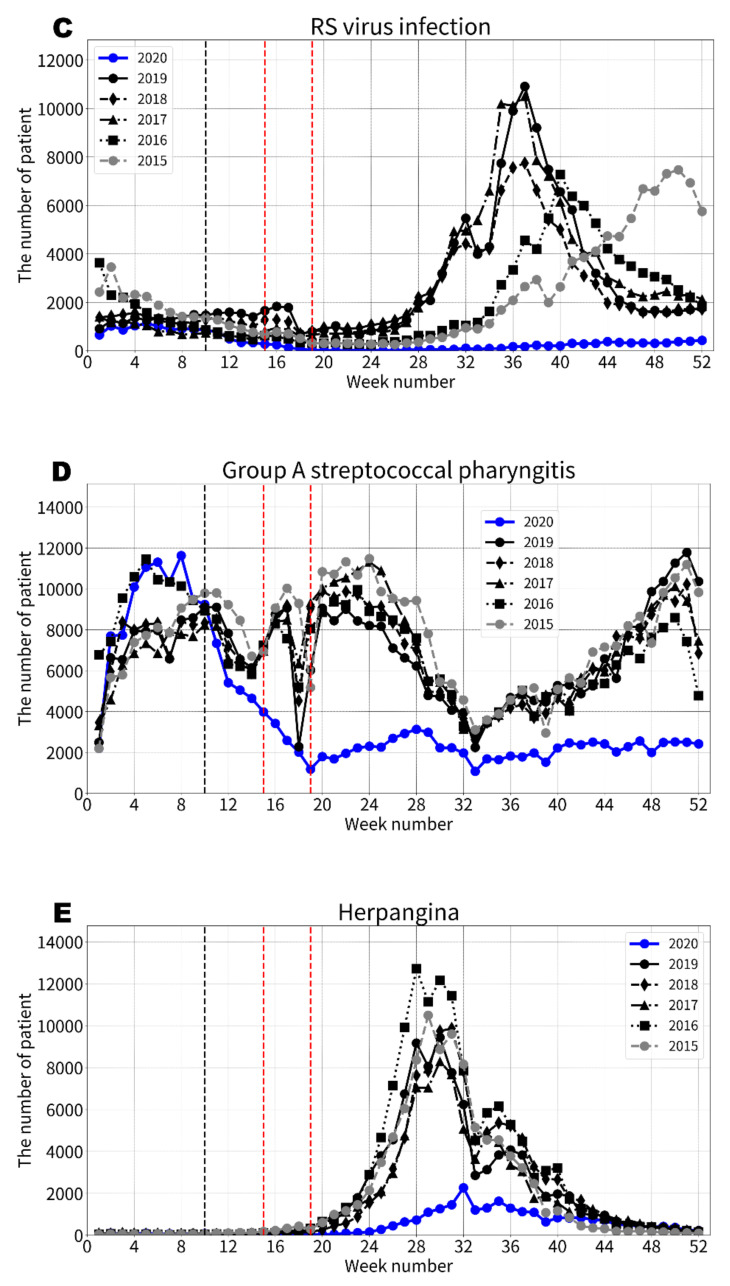
**The weekly trends in the numbers of patients with pediatric infectious diseases in the 1-year (52-week) period.** The initiation of the nationwide school closure at the 10th week is highlighted by the black dashed lines and the national State of Emergency from the 16th to 19th weeks is shown by the red dashed lines. The schools typically reopened after the State of Emergency was officially lifted in the 19th week. (**A**) Hand-foot-and-mouth disease. (**B**) Pharyngoconjunctival fever. (**C**) Respiratory syncytial virus infection. (**D**) Group A streptococcal pharyngitis. (**E**) Herpangina.

For all five specific PIDs, the numbers of patients identified after the 13th week in 2020 were consistently smaller than the minimum number of patients identified in 2015–2019 (Figure 2). The trends in the number of patients with PIDs differed according to the seasonal variation types. PIDs with a single peak in annual seasonality after the 20th week (HFMD, RSV infection, and herpangina) had consistently smaller numbers of infected patients in 2020 compared with the previous years without showing the peak trends in their annual epidemic seasons, although the number of patients with herpangina moderately increased during the peak season. The number of patients with herpangina identified after the 41st week in 2020 was similar to those identified during the same period in the previous years. Meanwhile, PIDs with multiple peaks in annual seasonality (PCF and GAS pharyngitis) showed different trends. Specifically, the number of patients with PCF and GAS pharyngitis identified in 2020 were similar to those identified in the previous years until the 10th week, and then dramatically decreased after the 11th week to much smaller numbers without subsequent increases during their peak seasons, although the number of patients with PCF showed a moderate increase in the peak season after the 41st week in 2020. 

**Figure 3:**
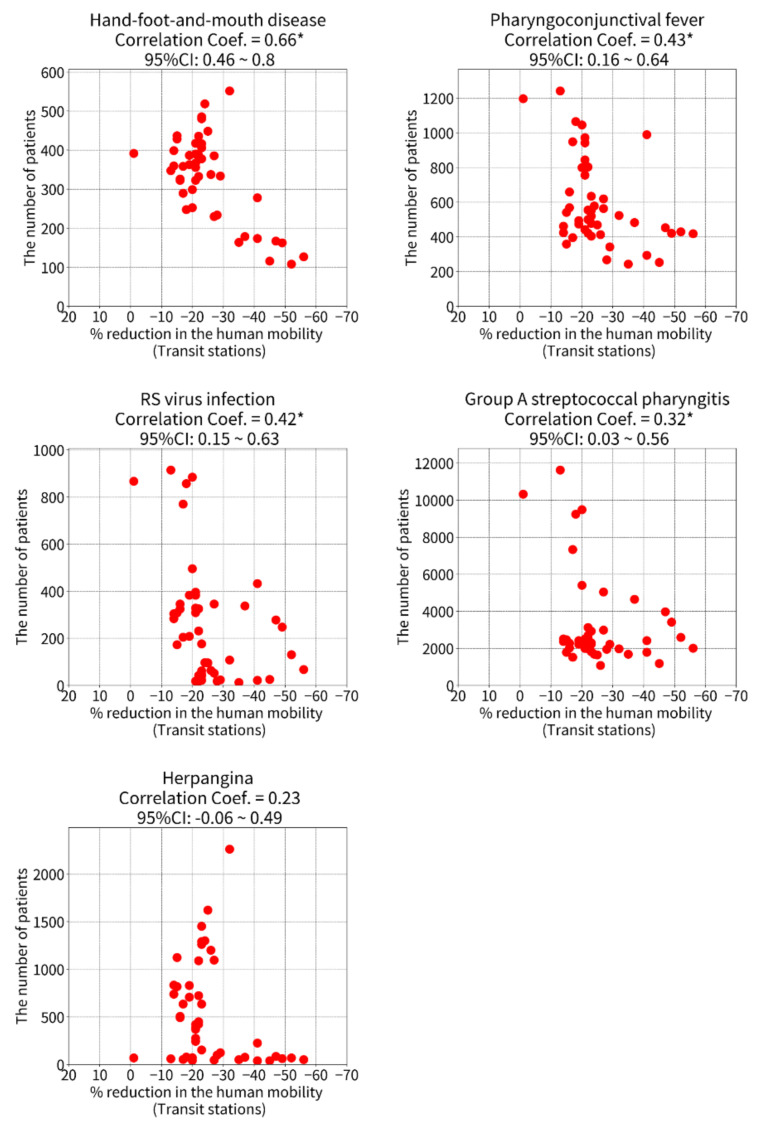
**The correlations between the number of patients with pediatric infectious diseases and the percentage reductions in human mobility at transit stations.** Fifty-two weeks of data (52 dots) in each scatter plot. The percentage reductions in human mobility negatively become larger when the movement of people is more greatly restricted. * Significant correlation (*p* < 0.05). Abbreviations: RS virus, respiratory syncytial virus; CI, confidence interval.

**Figure 4:**
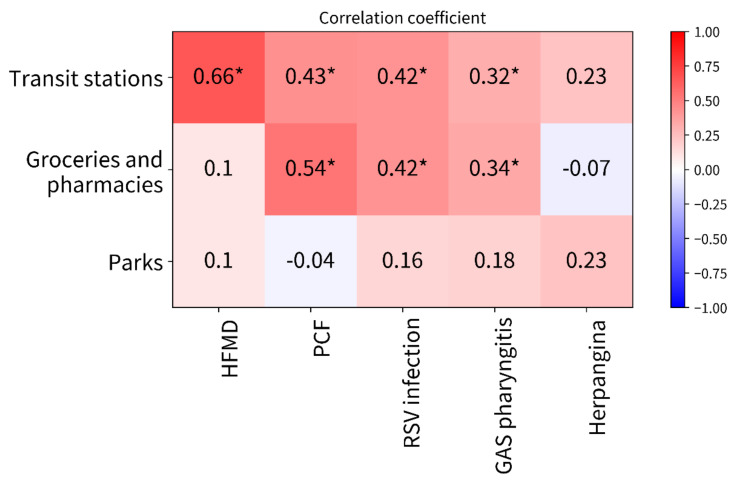
The heatmap of the correlations between the number of patients with pediatric infectious diseases and percentage reductions in human mobility. * Significant correlation. Abbreviations: HFMD, hand-foot-and-mouth disease; PCF, pharyngoconjunctival fever; RSV, respiratory syncytial virus; GAS pharyngitis: group A streptococcal pharyngitis.

Scatter plots show the associations between the percentage reductions in human mobility at transit stations and the number of patients with PIDs (Figure 3). Similar associations are shown in groceries and pharmacies (Supplementary Figure S1) and parks (Supplementary Figure S2). The correlation coefficients are summarized in Figure 3, which also shows a heatmap of the correlations between the number of patients with PIDs and the percentage reductions in human mobility in specific places. Among the PIDs, the Pearson correlation coefficients were highest for human mobility at transit stations, followed by groceries and pharmacies and then parks (Figure 4). Significant correlations were found between percentage reductions in human mobility at transit stations and the number of patients with HFMD (Pearson correlation coefficient [95% CI]: 0.65 [0.44–0.79]), PCF (0.47 [0.21–0.67]), RSV infection (0.45 [0.19–0.66]), and GAS pharyngitis (0.34 [0.06–0.58]). No significant correlations were found between the number of patients with PIDs and human mobility in parks.

## 4. Discussion

The present study provided four main findings. First, despite experiencing their peak seasons, dramatic reductions were observed in the number of patients with PCF and GAS pharyngitis after the 10th week in 2020 as prompt responses to the national school closure. Furthermore, the rapid decreases in human mobility in transit stations from the 14th week enhanced the reductions in the number of patients with PCF and GAS pharyngitis throughout 2020, with the smallest weekly numbers among the examined years since 2015. Second, no seasonal peaks after the 20th week were observed in the numbers of patients with all PIDs identified in 2020. In particular, the number of patients with HFMD and RSV infection, which have single seasonal peaks during the 30th–40th weeks in average years, were consistently minimized without any surge trends during the peak seasons. Third, the approximately 20% reduction in human mobility (at transit stations) appeared to have contributed to consistent reductions in the number of patients with PIDs. Finally, human mobility at transit stations had the highest correlations with the reduced number of patients with PIDs compared to those in groceries and pharmacies and parks. The heatmap chart suggested a trend that higher correlation values were found in places underlying potential human-to-human contacts among adults (transit stations > groceries and pharmacies > parks). To our knowledge, this is the first study that demonstrates that human mobility may be a potential factor affecting reductions in droplet-transmissible PIDs. 

In Japan, the government formally started to close schools nationwide from the 10th week in 2020. The school closure continued until the State of Emergency was officially lifted in the 19th week. During this period, nationwide schools were closed, and children were generally forced to stay home. However, unlike other global countries, citywide rigorous lockdown measures were not carried out in Japan. The number of patients with PIDs just in the middle of a peak season (PCF and GAS pharyngitis) were clearly reduced from the 10th week, coinciding with the start of the nationwide school closure. The number of patients with PCF and GAS pharyngitis were consistently decreased during the period of school closure (10th–19th weeks) with much smaller numbers than those identified in the previous years. Even after the 20th week when schools reopened throughout Japan, the number of patients with PCF and GAS pharyngitis identified in 2020 retained the large reductions and presented the smallest numbers since 2015. Furthermore, the number of children with HFMD, RSV infection, and herpangina identified after school reopening was also greatly reduced after the 20th week, even during the annual peak seasons. A study focusing on SARS-CoV-2 infection indicated that the number of children with COVID-19 did not rapidly increase after school reopening, while the easing of restrictions on large-scale gatherings had a major influence on rapid increases [27]. After the 20th week in Japan, the fact that people adhered to social distancing and standard infection control and prevention measures such as thorough hand-washing and mask-wearing, which may have resulted in the moderate increases in the number of patients with PIDs as well as COVID-19. A study conducted before the SARS-CoV-2 pandemic reported that hand-washing by children and their caregivers had a significant protective effect against community-acquired HFMD and herpangina [28]. Other studies have documented that increased compliance with social distancing measures can be a cost-effective strategy to mitigate the transmission of infectious diseases [29,30]. These studies support our explanations for the present findings.

The highest correlation coefficients were found for the associations between the number of patients with PIDs and human mobility at transit stations. The primary users of transit stations are typically adults. Therefore, the present findings indicate that greater reductions in the movement of adults with less potential human-to-human contacts may contribute to large decreases in the number of children with droplet-transmissible PIDs. For these reasons, our findings suggest that parent-to-child transmission may be a primary factor associated with increasing numbers of patients with PIDs, rather than cross-transmission among children. For example, RSV infection among children was greatly reduced without an annual peak season during 2020. Young infants are the dominant patients for RSV infection-associated hospital visits and hospitalizations [31]. Previous studies have indicated that parents most often introduced RSV into their households, leading to infection in infants [31,32,33,34], which could support our explanations for the present findings. The present results also suggested that an approximately 20% reduction in human mobility at transit stations (for adults) may contribute to the reduction in the number of patients with droplet-transmissible PIDs.

The study had some limitations. First, children with PIDs who lacked severe signs and symptoms would have refrained from visiting hospitals during the study period to avoid the potential risk for hospital-related asymptomatic transmission of SARS-CoV-2 [35], and this may have affected the decreased number of patients with PIDs identified throughout 2020. Second, the human mobility reports typically represent the activity of adults because the reports were based on data from Google users [24,25,26]. Therefore, the study did not include accurate information on the movements of children. Furthermore, the source population of human mobility at transit stations might differ from patients with PIDs. These were the limitations of the ecological study design. Finally, our data only reflected the weekly data on patients with PIDs from limited hospitals, although approximately 3000 hospitals throughout Japan participate in the surveillance of PIDs. The small sample comprising of only 52 weeks of data may have introduced statistical inaccuracy to the correlation coefficients between the number of patients with PIDs and human mobility.

## 5. Conclusions

We evaluated the associations between human mobility and trends in PIDs identified in 2020 in Japan. The highest correlations were found for the associations between the number of patients with PIDs and human mobility at transit stations, suggesting that adult-to-child transmission may be a primary factor associated with an increased number of patients with PIDs. A reduction in human mobility for adults may contribute toward decreases in the number of children with droplet-transmissible PIDs.

## Data Availability

Data were publicly available through internet websites. (1) National Institute of Infectious Disease, Japan. https://www.niid.go.jp/niid/en/data.html (accessed on 5 June 2022); (2) Ministry of Health, Labor and Welfare, Japan: Implementation Manual for the National Epidemiological Surveillance Infectious Diseases Program. https://www.mhlw.go.jp/english/policy/health-medical/health/index.html (accessed on 5 June 2022); (3) COVID-19 Community Mobility Reports. https://www.google.com/covid19/mobility/ (accessed on 5 June 2022).

## Supplementary Materials

The following supporting information can be downloaded at: https://www.mdpi.com/article/10.3390/ijerph19116941/s1, Figure S1: Correlations between the numbers of patients with pediatric infectious diseases and percentage reductions in human mobility at transit stations; Figure S2: Correlations between the number of patients with pediatric infectious diseases and percentage reductions in human mobility in groceries and pharmacies.
