# Supplementary material for: Human Mobility and Droplet-Transmissible Pediatric Infectious Diseases during the COVID-19 Pandemic
Source: Int J Environ Res Public Health. 2022 Jun 6;19(11):6941. doi: 10.3390/ijerph19116941 (PMC9180602; doi:10.3390/ijerph19116941)
Supplement: Supplementary file 1 [file ijerph-19-06941-s001.zip › ijerph-1709529-Figure S2.pdf]

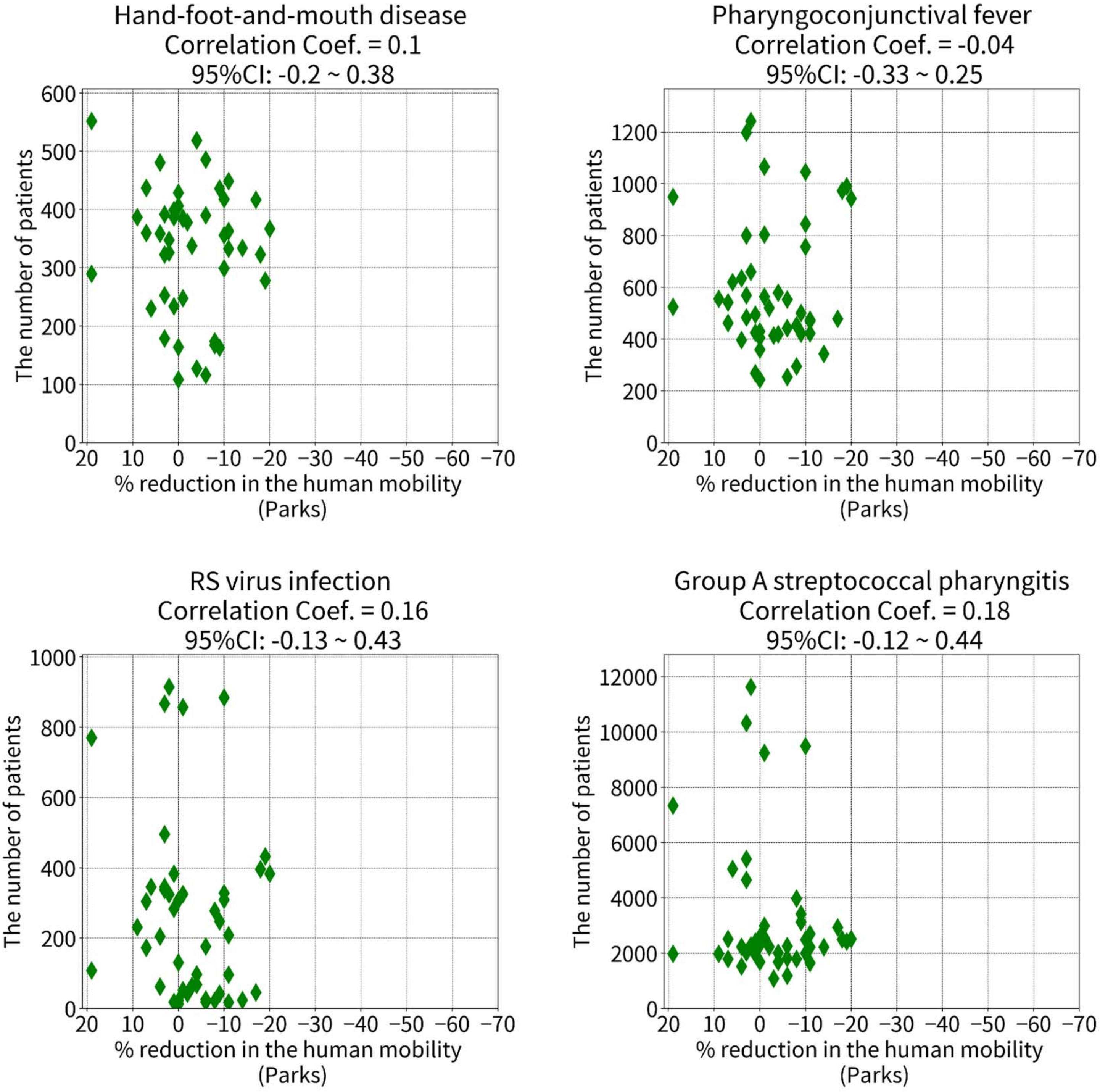

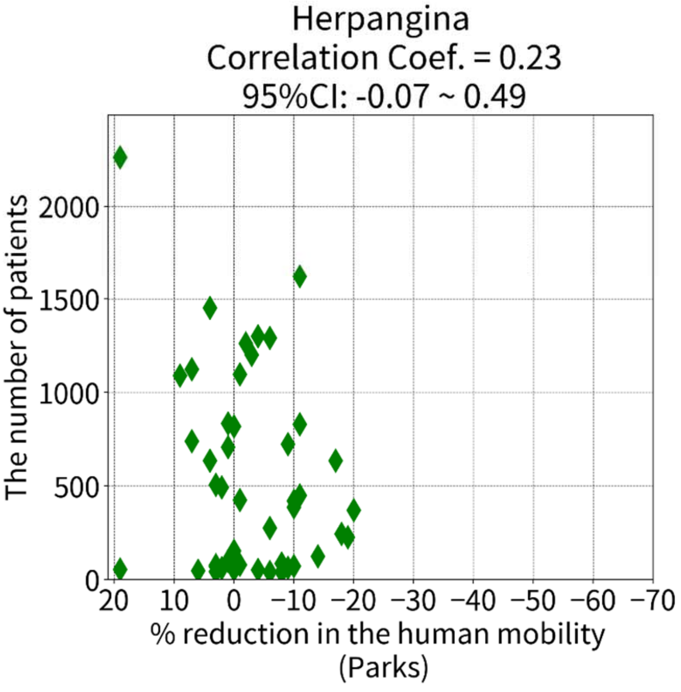

NOTE: *Significant correlation

Supplementary

Figure S2
